# Mediating effect of psychological safety on the relationship between inclusive leadership and nurses’ absenteeism

**DOI:** 10.1186/s12912-025-03464-4

**Published:** 2025-07-02

**Authors:** Ayman Mohamed El-Ashry, Bashair Mohamed Elsayed Abdo, Mahmoud Abdelwahab Khedr, Mona Metwally El-Sayed, Islam Sameh Abdelhay, Mennat-Allah G. Abou Zeid

**Affiliations:** 1 Psychiatric and Mental Health Nursing, Faculty of Nursing, Alexandria University, Alexandria, Egypt; 2 Department of Nursing Administration, Faculty of Nursing, Damanhour University, Beheira, Egypt; 3 Department of Nursing Administration, Faculty of Nursing, Mansoura University, Dakahlia, Egypt; 4 Nursing Administration Department, Faculty of Nursing, Ain Shams University, Cairo, Egypt; 5 College of Nursing, Prince Sattam Bin Abdulaziz University, Al-Kharj, Saudi Arabia

**Keywords:** Leadership, Absenteeism, Latent class analysis, Psychological safety, Nurses

## Abstract

**Objective:**

This study aimed to investigate the mediating role of psychological safety in the relationship between inclusive leadership and implicit absenteeism among nurses.

**Design:**

A descriptive, correlational cross-sectional design was conducted in accordance with the STROBE guidelines.

**Methods:**

Data were collected from 407 nurses working in two public hospitals in El-Behara Governorate, Egypt, using validated Arabic versions of the Inclusive Leadership Scale, Psychological Safety Scale, and the Stanford Presenteeism Scale (SPS-6). Descriptive statistics, Pearson’s correlations, multiple regression, and structural equation modeling (SEM) were employed to analyze the data using SPSS and AMOS (version 26).

**Results:**

Inclusive leadership was significantly and negatively associated with implicit absenteeism (*r* = − 0.207, *p* = 0.030) and positively associated with psychological safety (*r* = 0.204, *p* = 0.036). Psychological safety was also negatively correlated with implicit absenteeism (*r* = − 0.202, *p* = 0.041). Regression analysis revealed that both inclusive leadership (β = − 0.098, *p* = 0.049) and psychological safety (β = − 0.091, *p* = 0.048) significantly predicted lower implicit absenteeism. Furthermore, SEM results confirmed that psychological safety partially mediated the relationship between inclusive leadership and implicit absenteeism (indirect effect β = − 0.010). The model demonstrated an acceptable fit (RMSEA = 0.091; CFI = 1.000; IFI = 1.000; χ² = 9.748, *p* < 0.001).

**Conclusion:**

The findings highlight the critical role of inclusive leadership in reducing implicit absenteeism by fostering psychological safety. Promoting inclusive leadership practices and psychologically safe work environments may enhance nurse engagement, reduce presenteeism-related productivity loss, and ultimately improve patient care outcomes.

**Clinical trial registration:**

Not applicable.

## Introduction

Nurses constitute the largest group of healthcare professionals and are essential to the ongoing and comprehensive delivery of healthcare services [1]. On the other hand, because of their heavy workloads and complex work environments and objects, they frequently suffer from varied degrees of physical and mental health issues, which contribute to increased presenteeism or implicit absenteeism [2]. Implicit absenteeism is the phenomenon of people showing up for work despite having health issues that should cause them to take time off and rest. This leads to a decrease in focus and the inability to dedicate oneself entirely to one’s work, resulting in a decrease in productivity and an inability to execute duties [3]. Additionally, it impacts patient outcomes related to safety and health, the organization’s financial viability, and the standard of nursing care [4].

According to earlier studies, there is an expectation of implicit absenteeism in the workplace; rates range from 30% to over 90% across fifteen countries or regions [5]. Simultaneously, the negative consequences of employees’ implicit absenteeism have been thoroughly researched from several angles, such as impairment on employees’ health, decreased job satisfaction, and decreased productivity [6–8]. Healthcare workers were especially prone to implicit absenteeism [9], and due to their poor work habits, they would negatively impact patients, organizations, and themselves on several occasions [10].

## Inclusive leadership and implicit absenteeism

While implicit absenteeism has drawn the attention of academics from a wide range of disciplines, only a few studies have examined leaders’ impact on their subordinates’ implicit absenteeism from the standpoint of interpersonal interaction in the workplace [11]. There is strong evidence that inclusive leaders create a culture in their organizations that values unity, justice, and flexibility [12]. Inclusion places a strong focus on respecting, encouraging, and loyalty. This sets it apart from other ideas and approaches to management and Leadership [13]. Furthermore, inclusive leaders value their workers’ beliefs, encourage personal growth, and offer counsel through attentive listening. These numerous perspectives on inclusivity in leadership encourage workers to take responsibility and foster a climate that promotes task productivity, including task completion, psychological well-being, initiative, and creativity [14]. These advantages of having inclusive leaders should lessen nurses’ likelihood of being implicitly absent from work.

## Inclusive leadership and psychological safety

Psychological safety is “a common belief that taking interpersonal risks is safe within the team” [15]. A recent systematic review revealed that psychological safety has affected the psychological outcomes of healthcare providers. In particular, psychological safety reduces emotional fatigue and stress and increases job satisfaction, work engagement, organizational commitment, and empowerment. Moreover, psychological safety encourages healthcare providers to improve quality [16].

According to Amin et al. (2018), inclusive leadership is a new leadership style that can potentially improve patient safety [17]. Furthermore, a new study by Mikyoung and Moon (2019) showed a positive relationship between psychological safety, which bridges extra-role behavior by employees, and inclusive leadership and Inclusive Leadership [18]. Similarly, this article suggests that psychological safety may mediate between implicit absenteeism and inclusive leadership, reducing implicit absenteeism among staff members due to the supportive environment of psychological safety promoted by inclusive leadership.

However, there are 3 main gaps identified in the literature that warrant the conducting of the current study. First, insufficient Leadership-Centric Studies on Implicit Absenteeism. Most previous studies have focused on the personal, organizational, or structural factors contributing to implicit absenteeism but have neglected the role of leadership, especially inclusive leadership, as a potential solution to this issue [11, 12]. Second, while psychological safety has been linked to positive work outcomes, its role as a mediator in the relationship between leadership style and implicit absenteeism remains insufficiently explored. This study advances the field by investigating this indirect pathway, thereby providing a deeper understanding of the psychological mechanisms at play [15, 18]. Third, most of the existing research has been conducted in high-income countries, with little empirical work based on developing nations like Egypt. This study addresses the contextual gap by offering context-specific insights that may differ due to cultural, institutional, and organizational differences in healthcare delivery.

Additionally, this study adds novel insights to the small but expanding literature on inclusive leadership and its effect on nurses’ implicit absenteeism, with an emphasis on the Egyptian healthcare system, which has received less attention in international studies. Few studies have examined how leadership styles, particularly inclusive leadership, can either directly or indirectly ameliorate the negative consequences of implicit absenteeism on healthcare quality, employee well-being, and organizational productivity. Theoretical knowledge of these linkages is lacking because even fewer studies have used a theoretical lens to describe the underlying mechanisms.

## Theoretical framework

This study is grounded in the Conservation of Resources (COR) theory (Hobfoll, 1989), which posits that individuals are motivated to acquire, maintain, and protect valuable resources [19]. Gorgievski et al. (2011) state that everything that has value for someone might be categorized as a resource [20]. The central idea of COR theory holds that individuals try to get and preserve resources [21]. In high-demand work environments such as nursing, inclusive leadership serves as a critical job resource, offering emotional support, psychological empowerment, and meaningful interpersonal relationships. These resources can alleviate the psychological and physical strain that often leads to implicit absenteeism [22].

Through the lens of COR theory, inclusive leadership functions as a protective resource that replenishes nurses’ internal reserves. By fostering psychological safety, inclusive leaders create an environment in which staff feel valued, heard, and supported—conditions that mitigate the stress and burnout that contribute to working while unwell (implicit absenteeism). Therefore, the study proposes that psychological safety mediates the relationship between inclusive leadership and implicit absenteeism by reducing perceived resource loss and increasing resource gain.

## Research hypotheses

### H1

Inclusive leadership is negatively associated with implicit absenteeism among nurses.

### H2

Inclusive leadership is positively associated with psychological safety among nurses.

### H3

Psychological safety is negatively associated with implicit absenteeism among nurses.

### H4

**Fig. 1:**
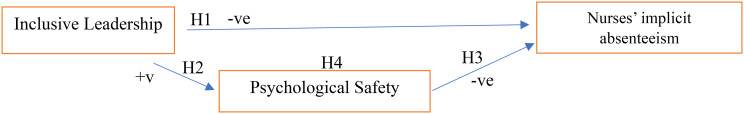
Study conceptual model

Psychological safety will mediate the relationship between inclusive leadership and implicit absenteeism among nurses (Fig. 1).

## Methods

### Study design

A descriptive, correlational cross-sectional study was conducted over three months from October 20, 2023, to the end of January 2024. The research adhered to the Strengthening the Reporting of Observational Studies in Epidemiology (STROBE) guidelines to ensure transparency and methodological rigor.

### Setting

The study was conducted in two public hospitals affiliated with the Egyptian Ministry of Health and Population in El-Behara Governorate: Damanhour General Hospital and Itay El-Baroud General Hospital. These hospitals serve urban and semi-urban populations and provide comprehensive inpatient and outpatient medical services. Damanhour General Hospital is a tertiary referral center and among the largest in the region, employing approximately 967 nurses and offering a wide range of specialized services across multiple departments. In contrast, Itay El-Baroud General Hospital has a smaller infrastructure, maintaining around 220 inpatient beds and employing an estimated 409 nurses. Data collection was conducted in three intensive care units (General ICU, Intermediate Care, and Emergency Care) and seven specialized medical departments (including General Medical Units for males and females, Pediatric, Isolation, Endoscopic, Hematemesis, Toxicology, and Dialysis units) at both hospitals. Recruitment was proportionally distributed according to hospital size and staffing levels to ensure balanced representation. Uniform data collection procedures, recruitment protocols, and ethical standards were applied across both hospitals to minimize potential institutional bias.

### Eligibility criteria

Eligible participants were registered nurses who held formal nursing qualifications, had taken at least one leave of absence in the past year, and voluntarily consented to participate in the study. Nurses currently in internship or training programs were excluded to ensure the sample reflected licensed professionals with relevant workplace experience.

### Sample size estimation

A convenience non-probability sampling technique was used to recruit participants. Sample size calculations were performed using G*Power software (version 3.1.9.4), applying a linear multiple regression model [23]. Based on a statistical power of 0.95, an alpha level of 0.05, and a medium effect size (f² = 0.15), as suggested by Cohen (2013) [24], and informed by prior studies (e.g., Gustafsson et al., 2020; Shan et al., 2021; Younas et al., 2020), the minimum required sample size was estimated to be 343 participants. Allowing for an anticipated 20% non-response rate, a total target of 407 nurses completed the survey.

### Recruitment process

**Fig. 2:**
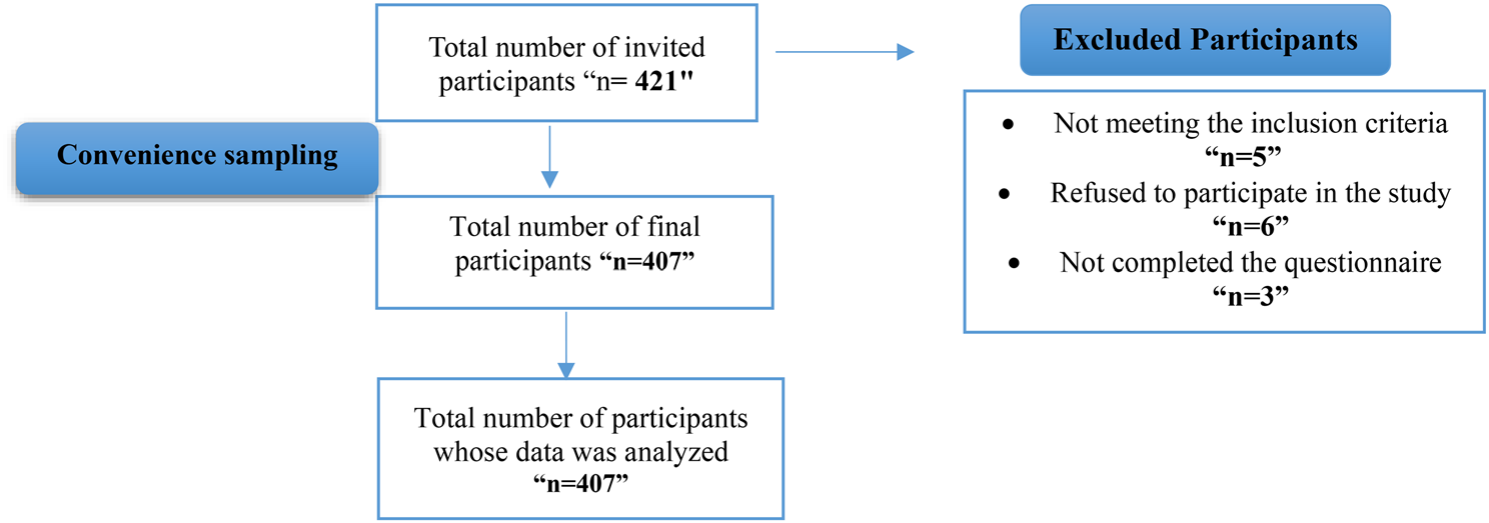
Participants’ recruitment flowchart

A total of 421 nurses were invited to participate in the study. Of these, 6 declined, 5 did not meet the eligibility criteria, and 3 withdrew after initially agreeing, resulting in a final sample of 407 participants, with a response rate of 95.5% and dropout rate of 4.5% (Fig. 2). Several factors may have contributed to this high response rate, including the direct relevance of the study to participants’ professional experiences and the voluntary nature of participation, which tends to attract motivated and engaged respondents. Additionally, the use of convenience sampling ensured accessibility and willingness to participate, further supporting robust recruitment outcomes.

### Study instruments

#### Nurses’ demographic characteristics profile

This profile was created to identify information about nurses’ age, gender, marital status, residence, nursing education, years of experience, working department, working hours, and family monthly income.

#### 10.1186/s12912-024-01841-zPsychological safety scale (PSS)

The Psychological Safety Scale, originally developed by Edmondson (1999), was adapted and translated for use in this study to assess the extent to which individuals perceive their work environment as safe for interpersonal risk-taking. The adapted version consists of seven items, each rated on a 5-point Likert scale ranging from 1 (strongly disagree) to 5 (strongly agree). Items capture aspects such as comfort in expressing concerns, admitting mistakes, and asking for help without fear of embarrassment or retribution. Total scores are calculated by summing responses across all items, with higher scores indicating a greater perception of psychological safety within the team or unit. To validate the Arabic version, confirmatory factor analysis (CFA) was conducted, which demonstrated an acceptable model fit: Comparative Fit Index (CFI) = 0.91, Tucker-Lewis Index (TLI) = 0.95, and Root Mean Square Error of Approximation (RMSEA) = 0.07. The internal consistency of the scale was robust, with a Cronbach’s alpha of 0.81, indicating good reliability. These psychometric properties support the use of the scale in evaluating psychological safety among nurses in Arabic-speaking healthcare contexts.

#### Inclusive leadership scale (ILS)

The Inclusive Leadership Scale (ILS) was employed to assess nurses’ perceptions of their leaders’ inclusive behaviors. Originally developed by Carmeli et al. (2010), the scale evaluates how leaders demonstrate openness, availability, and accessibility within the workplace. In this study, the validated Arabic version by Al-Atwi and Al-Hassani (2021) was utilized [25]. The ILS comprises nine items rated on a 5-point Likert scale, ranging from 1 (strongly disagree) to 5 (strongly agree). It captures three key dimensions of inclusive leadership: openness, which reflects the leader’s willingness to consider team members’ ideas and feedback; effectiveness, which indicates the leader’s competence and supportiveness; and amiability, which reflects approachability and interpersonal warmth. Higher scores on the scale indicate stronger perceptions of inclusive leadership. Confirmatory factor analysis (CFA) conducted on the Arabic version demonstrated a good model fit, with indices including CFI = 0.94, TLI = 0.96, and RMSEA = 0.06. Additionally, the scale showed strong internal consistency, evidenced by a Cronbach’s alpha of 0.86. These psychometric findings support the reliability and validity of the ILS for use in Arabic-speaking healthcare settings.

#### Stanford presenteeism scale – short form (SPS-6)

The Stanford Presenteeism Scale – Short Form (SPS-6) was utilized in this study to measure the extent to which nurses’ health problems interfered with their productivity over the past month. Developed by Koopman et al. (2002), the SPS-6 consists of six items that assess behaviors associated with implicit absenteeism, also known as presenteeism, where individuals attend work despite health issues that may hinder their performance [26]. Each item is rated on a 5-point Likert scale ranging from 1 (strongly disagree) to 5 (strongly agree), with total scores ranging from 6 to 30, where higher scores indicate greater levels of presenteeism or reduced productivity due to health-related issues. For the current study, the Arabic-translated version of the scale underwent confirmatory factor analysis (CFA) to assess its structural validity. The model demonstrated acceptable fit, with CFI = 0.91, TLI = 0.95, and RMSEA = 0.07, aligning with recommended psychometric thresholds. Additionally, the scale showed good internal consistency, with a Cronbach’s alpha of 0.83, indicating reliable measurement across items. These findings support the use of the SPS-6 as a valid and reliable tool for evaluating implicit absenteeism among nurses in Arabic-speaking healthcare contexts.

### Procedure

#### Ethical approval

The Nursing College at Damanhour University’s Research Ethics Committee (REC) has officially approved the study settings, allowing for necessary data collection. The responsible authorities in each hospital have also approved it. The study followed the Helsinki guidelines. Each study participant provided informed written consent after thoroughly explaining the study objectives. The study strictly adhered to voluntary participation, anonymity, and confidentiality. Additionally, study participants were informed of their right to withdraw from the study without any penalty.

#### Pilot study

A preliminary study was conducted to assess the study instruments for appropriateness, comprehensibility, and feasibility. Forty-five nurses who were not part of the primary study participated. The tools were found to be relevant, lucid, and practical, and no modifications or adjustments were deemed necessary.

### Data collection

A convenient sample was selected for this study, and data collection was carried out using self-administered questionnaires. The questionnaires were completed in a serene, private setting during the participants’ break at the hospital. The duration of the questionnaire completion was intentionally kept brief, between 10 and 15 min, to respect the valuable time of the nursing staff. This process emphasized the assurance of anonymity and confidentiality, reinforcing the ethical considerations of the study. Participation in the study was voluntary, with no incentives or penalties imposed for non-participation. To ensure the integrity and completeness of the data collected, each participant’s responses were meticulously reviewed. This rigorous approach aimed to minimize any potential gaps or inaccuracies in the data, thereby enhancing the reliability and validity of the study findings.

### Measures to reduce sampling bias

Several measures were taken to minimize bias associated with non-probability sampling and unequal hospital representation. Consistent inclusion and exclusion criteria were applied across both hospitals. Data collection procedures were standardized, with trained researchers following the same protocol. Recruitment was proportionally aligned with nurse staffing levels at each hospital to ensure balanced representation. Participation was anonymous and voluntary, reducing response bias. These steps enhanced the study’s internal validity and helped mitigate potential sampling bias.

### Data analysis

The study data were analyzed with IBM SPSS-26 and AMOS-26, involving review, coding, and cleaning processes. Normality was tested with Shapiro and Kolmogorov-Smirnov tests, while descriptive statistics and Pearson’s coefficient were used for participant characteristics and variable correlations. Cronbach’s Alpha assessed tool reliability and factor analysis validated translated instruments, with SPSS-AMOS used to calculate regression weights, standard error (SE), critical ratio (CR), and p-values. Goodness-of-fit measures like chi-square, root mean square error of approximation (RMSEA), comparative fit index (CFI), and Incremental Fit Index (IFI) were employed, with significance set at *p* < 0.01 for this study.

## Results

Table 1 shows the distribution of the studied nurses according to their socio-demographic characteristics. Regarding gender, the majority were female (80.1%), while males represented 19.9%. The mean age of the studied nurses was 27.05 ± 4.60 years. Marital status indicated that most were single (53.8%), followed by married individuals (44.7%). Family size mean numbers were 4.51 ± 1.54 family members. Educationally, a significant proportion held bachelor’s degrees (51.4%), followed by technical qualifications (36.9%). Work experience was primarily less than 5 years for all nurses. The weekly work hours mean score was 39.20 ± 9.84 h/ week. Health complaints ranged from skeletal problems (45.2%) to psychological problems (33.2%). The majority reported income sufficient to some extent (53.8%). Morning shifts were the most common (38.3%), followed by morning and night shifts (58.2%).

**Table 1:** Distribution of the studied sample according to socio-demographic characteristics (*n* = 407)

Demographic characteristics	No	%
**Gender**		
Female	326	80.1
Male	81	19.9
**Age**		
Mean ± SD	27.05 ± 4.60	
**Marital status**		
Single	219	53.8
Married	182	44.7
Divorced /Widowed	6	1.5
**Number of family members**		
Mean ± SD	4.51 ± 1.54	
**Level of education**		
Technical	150	36.9
BCs	209	51.4
Master	48	11.8
**Years of experience**		
0-<5	407	100
**Work hours per week**		
Mean ± SD	39.20 ± 9.84	
**Number of patients in your shift**		
< 10	376	92.4
10-<20	31	7.6
Mean ± SD	3.62 ± 5.18	
**Health complain**		
Hypertension/Hypotension	32	7.9
Skeletal problems	184	45.2
DM	6	1.5
Blood diseases	31	7.6
COVID	11	2.7
Cold and flu	8	2.0
Psychological problems	135	33.2
**Income**		
Sufficient	87	21.4
Sufficient to some extent	219	53.8
Insufficient	101	24.8
**Shifts**		
Morning	156	38.3
Night	14	3.4
Morning & Night	237	58.2

Table 2 presents the mean and mean percent scores for inclusive leadership, implicit absenteeism, and psychological safety among nurses. Inclusive leadership measures the degree to which nurses are open, effective, and amiable. The mean score for this scale was 28.94 ± 8.08, which translates to a mean percentage score of 55.70 ± 22.70. This suggests that, on average, the nurses exhibited a moderate level of inclusive leadership. The implicit absenteeism’s mean score was 18.71 ± 4.02, with a mean percentage score of 53.20 ± 16.77. This indicates that, on average, the nurses in this sample had a moderate level of implicit absenteeism. As well as psychological safety leadership measures the degree to which nurses create an environment where employees feel safe to take risks. The mean score for this scale was 18.29 ± 4.04, with a mean percentage score of 51.22 ± 16.82.

**Table 2:** Inclusive leadership scale, Stanford implicit absenteeism scale, and psychological safety scale (*n* = 407)

Study variables	Mean score	Mean percent
Inclusive Leadership	28.94 ± 8.08	55.70 ± 22.70
Openness subscale	9.92 ± 2.93	57.64 ± 24.41
Effectiveness subscale	12.79 ± 3.91	55.06 ± 24.47
Amiability subscale	6.23 ± 1.84	54.12 ± 23.29
Stanford Implicit Absenteeism	18.71 ± 4.02	53.20 ± 16.77
Psychological Safety	18.29 ± 4.04	51.22 ± 16.82

Table 3 displays Pearson correlation coefficients (r) and their significance (p) between study variables. According to the data, inclusive leadership and implicit absenteeism exhibit a significant negative correlation (*r*=-0.207, *p* = 0.030). On the other hand, psychological safety demonstrates a positive correlation with effectiveness (*r* = 0.207, *p* = 0.030) and inclusive leadership (*r* = 0.204, *p* = 0.036) and is negatively correlated with implicit absenteeism (*r*=-0.202, *p* = 0.041).

**Table 3:** Correlation between the studied variables (*n* = 407)

		Openness	Effectiveness	Amiability	Inclusive Leadership	Implicit Absenteeism
Openness	r					
	p					
Effectiveness	r	0.814*				
	p	< 0.001*				
Amiability	r	0.755*	0.777*			
	p	< 0.001*	< 0.001*			
Inclusive Leadership	r	0.929*	0.956*	0.878*		
	p	< 0.001*	< 0.001*	< 0.001*		
Implicit Absenteeism	r	-0.138*	-0.075	-0.092	-0.207*	
	p	0.005*	0.129	0.064	0.030*	
Psychological Safety	r	0.093	0.207*	0.081	0.204*	-0.202*
	p	0.061	0.031*	0.101	0.036*	0.041*

r: Pearson Correlation coefficient *: Statistically significant at *p* ≤ 0.05 (2-tailed)

Table 4 shows the results of a multivariate linear regression analysis on the factors affecting implicit absenteeism. The results indicate that inclusive leadership negatively affects implicit absenteeism, with a Beta of -0.098, a t-value of -1.976, and a p-value of 0.049. Similarly, psychological safety also negatively affects implicit absenteeism, with a Beta of -0.091, a t-value of -1.845, and a p-value of 0.048. The R2 value of 0.020 and the adjusted R2 value of 0.015 indicate that the model explains only a tiny proportion of the variance in implicit absenteeism. However, the F-value of 4.078 and the p-value of 0.018 indicated that the model is statistically significant at *p* ≤ 0.05.

**Table 4:** Multivariate linear regression analysis for factors affecting implicit absenteeism (*n* = 407)

Variable	B	Beta	t	*p*	95% CI	
					LL	UL
Inclusive Leadership	-0.050	-0.098	-1.976*	0.049*	-0.100	-0.001
Psychological Safety	-0.094	-0.091	-1.845*	0.048*	-0.193	0.006
R^2^ = 0.020, Adjusted R^2^ = 0.015, F = 4.078^*^, *p* = 0.018^*^						

F, p: f and p values for the model; R^2^: Coefficient of determination; B: Unstandardized Coefficients

Beta: Standardized Coefficients; t: t-test of significance; LL: Lower limit UL: Upper Limit

*: Statistically significant at *p* ≤ 0.05

**Fig. 3:**
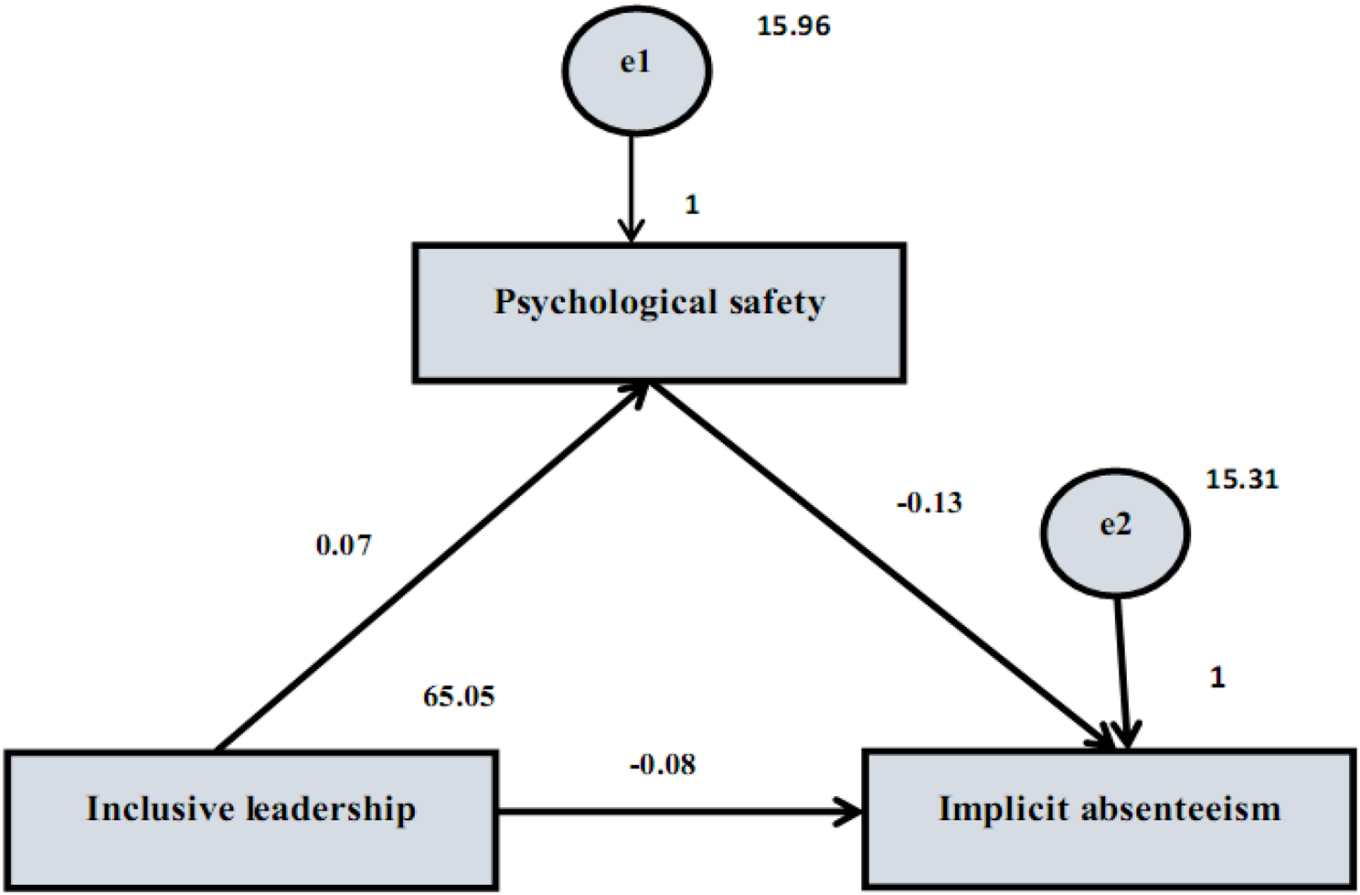
Path analysis to detect the Direct and Indirect effects of the Inclusive Leadership Scale on the Stanford Presenteeism Scale, mediating by Psychological Safety Leadership

The direct and indirect effects of implicit absenteeism, inclusive leadership, and psychological safety are shown in Table 5; Fig. 3. Inclusive leadership had a direct effect of 0.073 on psychological safety, which means that holding other factors equal, a rise of one unit in the former corresponds to a 0.073 unit increase in the latter. An effect is considered statistically significant with a p-value of 0.003, less than 0.05. Likewise, the regression coefficient between implicit absenteeism and inclusive leadership was − 0.080, meaning that for every unit increase in the former, there was a 0.080 decrease. All other variables remained constant. The p-value of less than 0.001 further confirmed the importance of this impact. When all other factors are held constant, the direct effect of psychological safety on implicit absenteeism was − 0.130, indicating a 0.130 unit drop in the latter for every unit rise in the former. Because the p-value was less than 0.001, the statistical significance of this impact was confirmed. At -0.010, psychological safety mediated the connection between implicit absenteeism and inclusive leadership. The model showed satisfactory fit parameters with RMSEA of 0.091 (acceptable) and CFI and IFI of 1.000 (strong fit). The model’s chi-square value was significant (9.748, *p* < 0.001), validating its fit to the observed data.

**Table 5:** Direct and indirect effect

Variable 1		Variable 2	Direct effect	Indirect effect	C.*R*	*p*-value
**Psychological Safety**	←	**Inclusive Leadership**	0.073	0.0	2.997*	0.003*
**Implicit Absenteeism**	←	**Inclusive Leadership**	-0.080	-0.010	-3.292*	< 0.001*
**Implicit Absenteeism**	←	**Psychological Safety**	-0.130	0.0	-2.673*	< 0.001*

Model fit parameters CFI; IFI; RMSEA (1.000; 1.000; 0.091)

CFI = Comparative fit index; IFI = incremental fit index; and RMSEA = Root Mean Square Error of Approximation. Model χ^2^; significance 9.748^*^(< 0.001^*^)

## Discussion

Healthcare workers must operate in demanding, fast-paced workplaces where making precise decisions, minimizing errors, and devising creative solutions are crucial to delivering top-notch patient care [27]. Psychological safety creates an environment where individuals feel comfortable being who they are. Enhancing employee voice, dedication to the company, and financial support for patient care are ways to do this [28]. This study provides an in-depth understanding of psychological safety among critical care nurses. It aimed to identify the mediating role of psychological safety between inclusive leadership and implicit absenteeism among nurses.

According to the study, the nurses demonstrated modest implicit absenteeism, inclusive leadership, and psychological safety. Implicit absenteeism manifests when nurses report to work while unwell, leading to decreased focus and engagement. This phenomenon can stem from factors such as chronic stress, heavy workloads, and organizational culture that discourages taking leave [9]. A helpful and inclusive work atmosphere is created by inclusive leadership, which promotes psychological safety among nurses. Employees are more willing to speak honestly, offer suggestions, and deal with problems about their well-being when they feel psychologically comfortable, eventually lowering implicit absenteeism. This is consistent with earlier studies that highlight the value of inclusive leadership in fostering fruitful work environments [29, 30]. According to research, inclusive leadership is essential in fostering a welcoming and inclusive workplace, which is critical for nurses’ productivity and well-being [31].

It has been shown that leadership, incredibly inclusive leadership, helps nurses get over psychological pain and decreases their desire to quit. According to Carmeli et al. (2010), inclusive leadership exhibits Effectiveness, accessibility, and openness while working with subordinates [32]. The results of this study demonstrated a strong negative correlation between Implicit Absenteeism and Inclusive Leadership. Recent studies have shown that implementing inclusive leadership strategies may increase presenteeism reduction and employee productivity, resulting in a more productive and successful workplace. These results are consistent with earlier research by Javed et al. (2019) that showed a favorable relationship between creative work practices, inclusive leadership styles, and psychological empowerment [33]. Furthermore, studies have demonstrated that inclusive leadership may improve psychological safety, promote a calm frame of mind, and lessen psychological discomfort among nurses [34, 35].

While previous research provides valuable insights into the benefits of inclusive leadership, it is important to acknowledge that not all studies uniformly support these conclusions. Some literature suggests that inclusive practices may not be universally effective and can depend on contextual factors, such as team dynamics and individual differences among staff [36, 37]. Moreover, the implications of implicit absenteeism on patient care are significant. When nurses are implicitly absent, they may be less attentive and more prone to errors, ultimately compromising patient safety and care quality [8, 9]. Understanding the root causes of implicit absenteeism is crucial for addressing this issue effectively, as unresolved stressors can perpetuate the cycle of absenteeism and dissatisfaction among nursing staff.

Enhancing psychological safety in the workplace has been linked to better job performance in various professions. Organizations may improve psychological safety by taking several steps, including breaking down hierarchical systems, encouraging transparency, ensuring employees are easily accessible, and establishing trust [38, 39]. When nurses feel psychologically safe at work, they are more likely to communicate openly, have more job satisfaction, have a lower intention to leave, and have better patient safety [40]. In this respect, the current study results revealed that psychological safety positively correlates with the inclusive leadership scale and negatively correlates with implicit absenteeism. This may be attributed to the fact that when nurses perceive their leaders as inclusive and supportive, they are more likely to feel psychologically safe in their work environment. Inclusive leaders foster a sense of belonging, encourage open communication, and value diverse perspectives, which creates a psychologically safe climate [41]. This, in turn, reduces employees’ fear of negative repercussions for expressing concerns, making mistakes, or seeking help, thereby promoting their engagement and commitment to work. Nurses who feel psychologically safe are more likely to be present and actively engaged in their roles, leading to lower levels of implicit absenteeism.

Research evidence supports the current mediating role of psychological safety. For instance, a study by Lee and Dahinten (2021) found that an inclusive leadership style improved the psychological safety of nurses [35]. It was also found that nurses’ psychological safety must be prioritized and enhanced to improve patient safety and decrease turnover. As a result, nurses will feel more empowered to speak up and point out errors when they work under inclusive leaders. Similarly, another study done by Wang et al. (2021) identified that psychological safety could improve nurses’ performance [42]. In a psychologically safe environment, genuine feedback is enabled and encouraged, collaboration is genuinely cultivated, and there is a willingness to experiment with new strategies, even when the consequences are undefined [43]. Associated consequences of psychological safety in healthcare include improved staff well-being, decreased stress, and devotion to improving quality [27, 44].

Improved psychological safety in healthcare settings can lead to enhanced staff well-being, decreased stress, and a strong commitment to quality improvement [37]. Addressing the issues surrounding implicit absenteeism through inclusive leadership and a focus on psychological safety can lead to better outcomes for both staff and patients.

### Study limitations

Utilizing a non-probability convenience sampling method in the study might result in sampling bias and restrict the applicability of the results to the larger nursing population. Differences in characteristics between participants and non-participants or ineligible individuals could impact the findings. The cross-sectional design of the study offers a momentary view of the data. It does not facilitate the establishment of causal links or the observation of changes over time, while the research’s restriction to two specific hospitals in one region may hinder its generalizability to other healthcare environments with varying systems, cultures, or socioeconomic factors. Despite attempts to manage confounding variables, undisclosed variables could affect the relationships among the variables under investigation.

## Conclusion

The relationship between inclusive leadership and implicit absenteeism among nurses is complex, and psychological safety plays a crucial role in mediating this relationship. Inclusive leadership fosters a work environment that is supportive, inclusive, and psychologically safe, which, in turn, reduces implicit absenteeism among nurses. Therefore, healthcare organizations must prioritize developing and implementing inclusive leadership practices to enhance psychological safety and mitigate the negative impact of implicit absenteeism on nurses. Organizations can improve employee well-being, retention, and overall patient care quality by creating a work environment that values inclusivity and psychological safety. Ultimately, this can lead to enhanced productivity and organizational outcomes.

### Implications for nursing and health policy

Nurses play a crucial role in delivering quality patient care. Inclusive leadership can help create a supportive work environment that fosters psychological safety, job satisfaction, and patient safety. Nurse leaders should promote open communication and encourage feedback to ensure a psychologically safe environment. Organizations should address implicit absenteeism, which negatively impacts nurse productivity and patient care, by prioritizing employee well-being and fostering inclusive leadership. Nurses must understand the relationship between psychological safety, nurse well-being, and patient safety, and actively work to prioritize psychological safety to enhance patient care quality [45, 46].

Health policy should promote inclusive leadership and psychological safety in healthcare organizations, supporting the creation of supportive work environments for nurses. Government agencies should mandate psychological safety measures in healthcare settings, including inclusive leadership training and protocols for workplace issues [47, 48].

To implement these findings practically, healthcare organizations should consider developing specific leadership training programs focused on inclusive practices. These programs can equip nurse leaders with the skills to foster a culture of psychological safety by enhancing their communication and feedback strategies. Additionally, organizations may benefit from implementing regular assessments of workplace culture and employee well-being to identify areas for improvement. Health policy should also monitor and evaluate psychological safety initiatives in healthcare and offer incentives to organizations that prioritize psychological safety and inclusive leadership. Concrete strategies such as establishing mentorship programs for nurses, creating safe spaces for open dialogue, and developing clear policies for addressing workplace concerns can further enhance psychological safety. Collaboration among stakeholders is encouraged by health policy to promote psychological safety in nursing, ensuring that both nurses and patients benefit from a healthier workplace environment.

## Data Availability

Data will be available upon reasonable request from the corresponding author.
